# Achieving Strength–Ductility Balance in TWIP Steel by Tailoring Cementite

**DOI:** 10.3390/ma18040843

**Published:** 2025-02-14

**Authors:** Zhenyu Zhao, Jian Sheng, Dazhao Li, Shaobin Bai, Yongan Chen, Haitao Lu, Pengfei Cao, Xin Liu

**Affiliations:** 1 School of Materials Science and Engineering, North University of China, Taiyuan 030051, China; 13903402492@163.com (Z.Z.); shengjian032@163.com (J.S.); cya894589913@163.com (Y.C.); c17824944905@163.com (P.C.); 13935140840@163.com (X.L.); 2 Shanxi Key Laboratory of Advanced Metal Materials for Special Environments, Taiyuan 030051, China; 3 School of Aerospace Engineering, North University of China, Taiyuan 030051, China; 4 School of Materials Science and Engineering, Taiyuan University of Science and Technology, Taiyuan 030024, China; b202314110015@stu.tyust.edu.cn

**Keywords:** high-Mn TWIP steel, cementite particle, recrystallization, deformation mechanism, strength–ductility balance

## Abstract

High-Mn steels are widely used in various fields. However, the FCC structure is not conducive to improving strength, limiting their development and application. In this work, hot-rolled Fe-25Mn-1Al-3Si-1C (wt.%) steel was annealed at various temperatures to tailor the cementite particles and recrystallized grains, thus achieving a balance between strength and ductility. As the annealing temperature increased from 550 to 650 °C, the volume fraction of recrystallized grains slightly increased and the volume fraction of cementite particles initially increased and then decreased, which was explained and verified by the quantitative calculation. Especially, the high-density pre-dislocation and finely dispersed cementite particles in sample AN550 resulted in a relatively low volume fraction of recrystallized grains. Interestingly, secondary deformation twinning was activated during the subsequent tensile deformation in addition to the dislocations, stacking faults, and previous deformation twinning. This complex interaction among various deformation mechanisms indued a good balance between strength and ductility, achieving an outstanding result (58.9 GPa%) regarding tensile strength and total elongation. This work offers an effective route for developing a high-Mn TWIP steel with outstanding strength–ductility balance.

## 1. Introduction

In recent years, TWIP (twinning-induced plasticity) steel has gained extensive recognition due to its favorable product of strength and elongation (PSE). It is considered a crucial candidate material in the automotive industry. The excellent PSE of steel is mainly attributed to its ductility [1,2,3,4]. The large ductility of TWIP steel is explained by its single-face-centered cubic microstructure, because numerous slip systems can be activated, and deformation twinning can be achieved during plastic deformation [5]. However, an increase in ductility will inevitably lead to a decrease in strength. Therefore, it is important to optimize the strength–ductility balance of TWIP steel, enhancing its strength.

Numerous scholars have employed various approaches to enhance the strength of TWIP steel. The yield strength of steel can be enhanced through the use of dislocation, fine grains, precipitates, etc. [6]. Early work on the micro-alloying of TWIP steels was employed to enhance strength via solid solution strengthening and the Orowan precipitate bypassing mechanism [7]. Liang et al. [8] enhanced the yield strength (YS) of high-Mn steel through Nb micro-alloying. However, this method not only resulted in high costs but also increased the difficulty of smelting [9]. Some researchers improved mechanical properties by introducing nano-scale precipitates. Qiu et al. [5] employed a cold-roll annealing process to refine the grains and introduce a uniform distribution of nano-scale precipitates (κ-carbide), which interact with the dislocation and effectively improve the strength of high-Mn steel. However, the cold-rolling process leads to a dislocation density approaching saturation, limiting continuous work hardening during deformation, thus leading to a significant deterioration in ductility [5]. Furthermore, the cold-rolling process has drawbacks, such as high energy consumption and complex procedures. The hot-rolling process requires lower rolling forces and offers a lower cost. In addition, the surface quality and strength of hot-rolled steel plates have been improved significantly in recent years [9]. Studies have shown that the combination of nano-scale precipitates and dislocations may reduce the mutual limitations in terms of strength and ductility [5]. At the same time, the introduction of partially recrystallized microstructures is conducive to improvements in ductility [10]. Hu et al. [11] pointed out that the precipitation of nano-scale precipitates has a significant impact on recrystallization behavior via solute drag and Zener pinning effects. In turn, recrystallization interacts with precipitates, thereby influencing mechanical properties. Ren et al. [12] reported that tailoring the precipitation of nano-scale carbides via the hot-rolling–annealing process and mixing the recrystallization structure can improve strength while retaining ductility. The results show that this is an effective method with which to increase strength by introducing carbides and recrystallization structures via the hot rolling-annealing process. However, the influence of nano-scale carbides on recrystallization behavior remains unclear during annealing. In addition, the existence of precipitates, dislocations, partial recrystallization, and other microstructures results in complex deformation mechanisms. Meanwhile, the reaction mechanism of competition and coupling during plastic deformation remains unclear.

In this work, the hot-rolling process is employed to refine grains and introduce high dislocation densities in Fe-25Mn-1Al-3Si-0.2C (wt.%) steel. Then, carbide precipitation is tailored by controlling the annealing temperature and achieving an excellent balance between strength and toughness. The PSE value reaches 58.9 GPa%. Subsequently, the influence of precipitates on recrystallization behavior is discussed. Moreover, the evolution behavior of microstructure and various deformation mechanisms during tensile deformation are investigated.

## 2. Experimental Section

**Figure 1:**
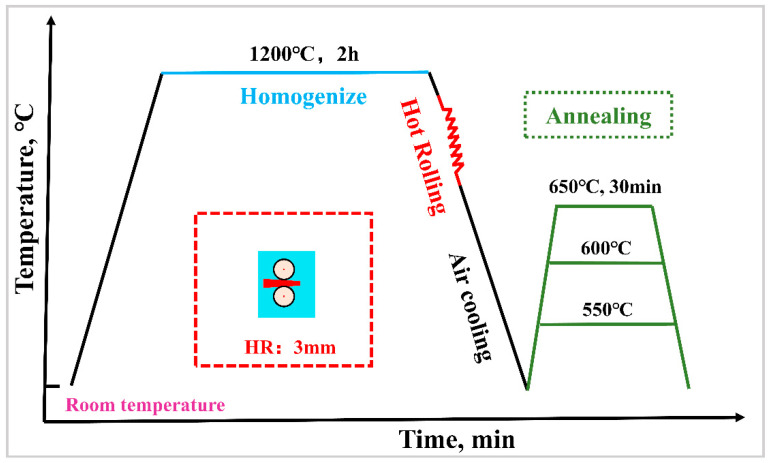
Abstract process flow diagram.

The composition of the TWIP steel used in the experimental methods is Fe-25Mn-1Al-3Si-0.2C (wt.%). This was melted in a 50 kg vacuum induction furnace and then cast into a mold with dimensions of Ø170 × 350 mm. After homogenizing at 1200 °C for 2 h in an Ar atmosphere, the ingot was hot-rolled into 3 mm plates in 7 passes along the axial direction, with initial and final forging temperatures of 1150 °C and 900 °C, respectively. Large deformations were applied during the hot-rolling process to induce high dislocation densities and fine grains. After hot rolling, the specimens were cut from the rolled plate and then subjected to a modulated annealing treatment in an Ar atmosphere at 550 °C, 600 °C, and 650 °C for 30 min, respectively. The test samples were designated as HR (hot-rolled state), AN550, AN600, and AN650. The process flow diagram is shown in Figure 1.

The microstructures of the specimens were characterized by X-ray diffraction (XRD, SmartLab-3 KW), scanning electron microscopy (SEM), electron backscatter diffraction (EBSD), and transmission electron microscopy (TEM). To determine the existing phases, X-ray diffraction was performed by scanning at 40 kV with a current of 200 mA using CuKα radiation in the 2θ range of 20° to 100° at a scan rate of 5°/min. The microstructure was observed in the transverse direction (perpendicular to the rolling surface) using a field-emission SEM (Tescan Mira3, TESCAN Group, Shanghai, China) equipped with an EBSD system. The SEM-EDS result was analyzed by using EDAX TEAM. The SEM and EBSD samples were first mechanically polished and thereafter electro-polished for 35–40 s using 10% perchloric acid and 90% ethanol electrolyte at 15.8 V and −20 °C. The EBSD scanning step size was 0.4 μm, and the size of the scanned are was 291.6 µm × 223.69 µm. The data were analyzed utilizing OIM7.3 software. Cleaning procedures were implemented. We appropriately employed the Grain CI standardization cleanup method. For this method, the parameter minimum grain size was set to 1, and the parameter grain tolerance angle was set to 5 in OIM7.3 software. The TEM sample was initially mechanically ground to a thickness of 50 μm and then punched into 3 mm diameter disk. Subsequently, a twin-jet electric polisher was employed for further thinning in a solution containing 5% perchloric acid and ethanol. Finally, TEM observation was conducted using the JEM-2100F (JEOL, Tokyo, Japan) field-emission electron microscope at an acceleration voltage of 200 kV. The tensile samples, 70 mm long and 6.0 mm wide, were made by electrical discharge machining (EDM) of the hot-rolled plate along the rolling direction. Tensile testing was performed on a UTM 5305 tester with a strain rate of 10^−3^ s^−1^. Tensile experimental data were obtained three times for each sample, and the average value was determined.

## 3. Results

### 3.1. Microstructure Characterization

**Figure 2:**
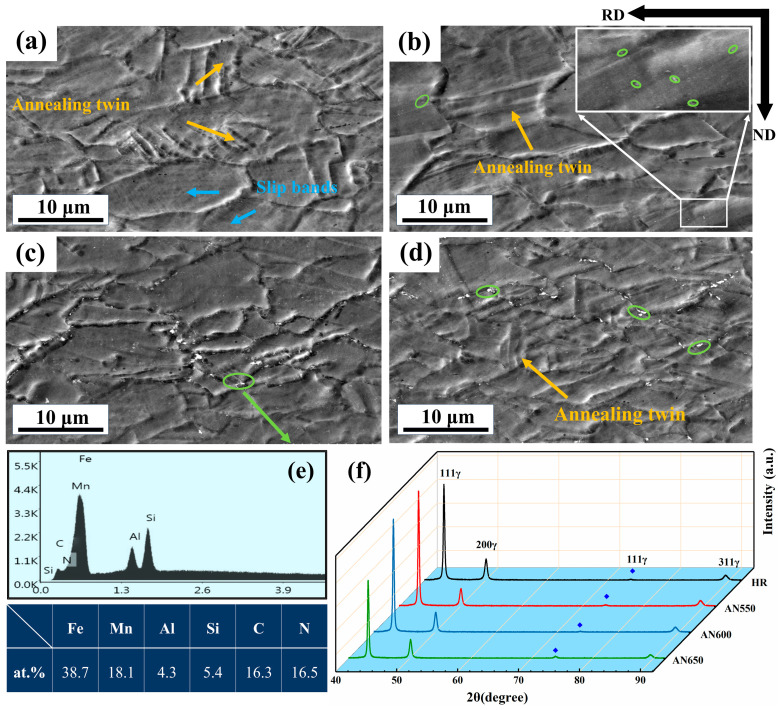
SEM microstructures of samples: (**a**) HR; (**b**) AN550; (**c**) AN600; (**d**) AN650. (**e**) EDS result of cementite particles in sample AN600. (**f**) XRD spectra of samples under various conditions.

Figure 2 shows the microstructures of samples under various conditions. In the hot-rolled sample, a large number of annealing twins and slip bands were observed in austenite grains (Figure 2a). However, no significant precipitates were observed (Figure 2a). Figure 2b–d show the microstructures of samples AN550, AN600, and AN650, respectively. For all annealed samples, the annealing twins and precipitates are present within austenite grains and along grain boundaries, respectively. In contrast, the precipitate size in sample AN550 was much smaller than in other samples. As shown in the enlarged image in Figure 2b, numerous fine precipitates were intra-granular in addition to the grain boundaries (GBs). For samples AN600 and AN650, the increase in annealing temperature inevitably induced the coarsening of precipitates (green ellipses), especially the particles in grain boundaries. Figure 2e shows the EDS result of the precipitate in sample AN600. Its chemical composition was 38.7Fe-18Mn-16.3C (at%), implying that similar precipitates included (Fe, Mn)_3_C cementite particles. Figure 2f displays the XRD results of samples before and after annealing. It can be seen that only single γ-Fe diffraction peaks were exhibited, indicating a fully austenitic single phase without any phase transformation.

**Figure 3:**
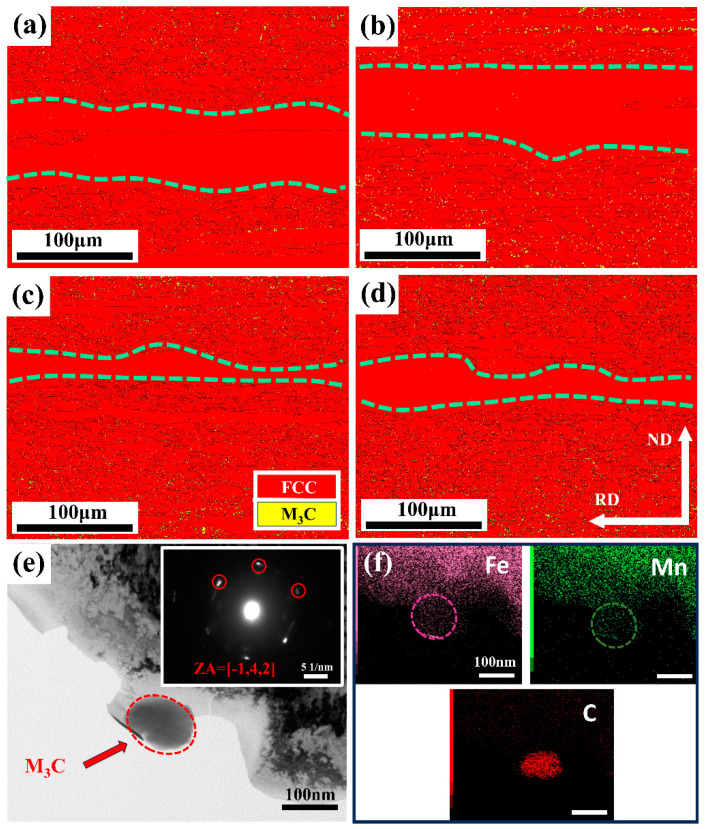
EBSD and TEM micrographs of samples under various conditions. (**a**–**d**) The phase diagrams of coexisting austenite and precipitate: HR, AN550, AN600 and AN650, respectively. (**e**) shows the precipitates observed in the AN550 sample, and (**f**) is the EDS map of the red region of (**e**).

EBSD and TEM were performed to analyze the microstructures (Figure 3). Banded structures (dashed lines) and precipitates (M_3_C) were present within the austenitic matrix. These banded structures were formed due to the uneven distribution of Mn, which also affected recrystallization behavior [13]. For the hot-rolled sample, a small number of fine precipitates were detected (Figure 3a). In contrast, there were many more nano-scale particles exhibited in AN550 samples (Figure 3b). TEM-EDS mapping (Figure 3f) clearly showed that Fe, Mn, and C were enriched in the precipitates. The corresponding selected area diffraction pattern (SADP) verified these nano-scale particles were (Fe, Mn)_3_C cementite (Figure 3e). As the annealing temperature increased, the fine precipitates within grains gradually dissolved, while the precipitates distributed along GBs gradually coarsened (Figure 3c,d).

**Figure 4:**
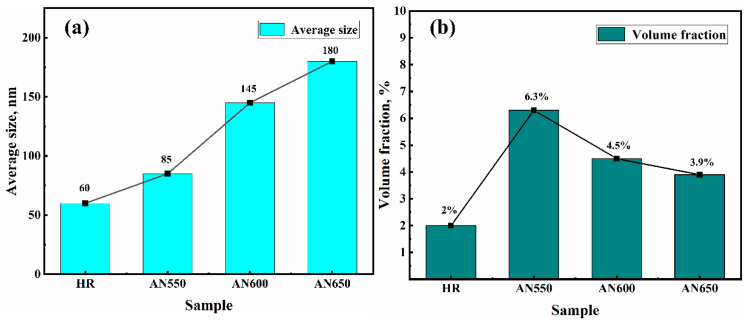
(**a**,**b**) are the volume fraction and average grain size of the carbides, respectively.

This finding was highly consistent with the SEM results in Figure 2. More than five different SEM images of the sample were used to measure the volume fraction and average size of cementite particles using Image-Pro software6.0. The volume fraction (*f*) and average size (*x*) of cementite particles were statistically measured (Figure 4).

**Figure 5:**
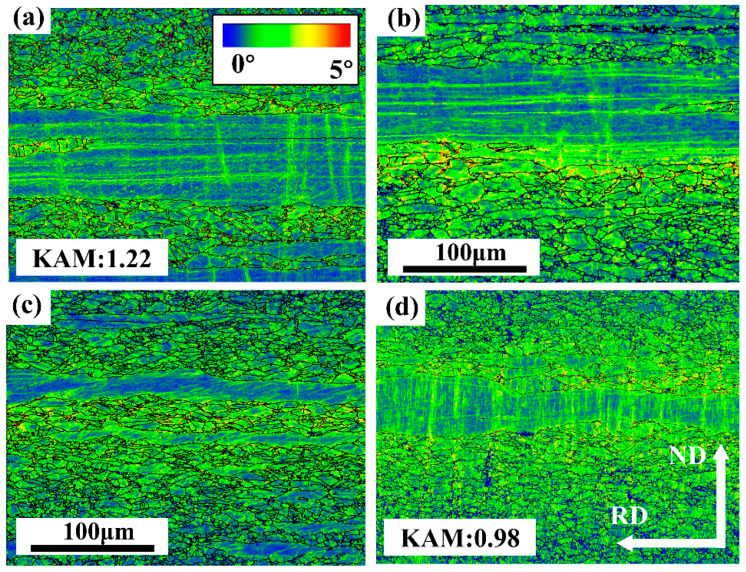
KAM maps of the samples under various conditions. (**a**) HR, (**b**) AN550, (**c**) AN600, (**d**) AN650. 0–1°, recrystallized grains; 1–5°, non-recrystallized grains.

Figure 5a–d show the kernel average misorientation (KAM) maps and the corresponding KAM values of all samples. The KAM value can reflect the geometric dislocation density and recrystallization volume fraction of the sample [13,14,15,16]. The KAM value decreased from 1.22 to 0.98 with the increased annealing temperature. It can be seen that the recrystallized volume fraction and the dislocation density increase and decrease with the increase in annealing temperature, respectively.

**Figure 6:**
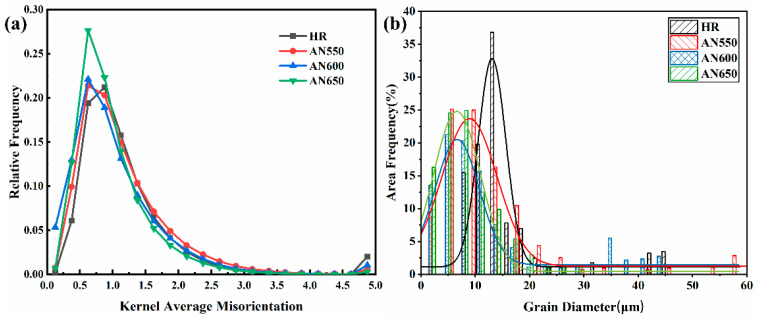
(**a**) KAM distribution map of the sample. (**b**) Average grain size distribution of the sample under various conditions (excluding Mn segregation band).

The specific KAM values calculated are shown in Figure 6a. Figure 6b shows the statistical distribution of grain sizes for all samples, which were fitted with a normal distribution function and are summarized in Table 1. It can be seen that the grain size decreases with the increase in annealing temperature.

### 3.2. Mechanical Properties

**Figure 7:**
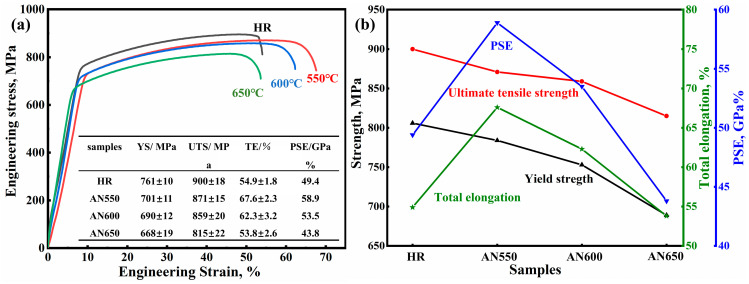
Mechanical responses at different annealing temperatures. (**a**) Engineering stress–strain curves; (**b**) the tensile mechanical properties of samples in terms of yield strength, ultimate tensile strength, total elongation, and UTS × TE.

Figure 7a,b show the engineering stress–strain curve and the measured mechanical properties of all samples. As the annealing temperature increased, the yield strength (YS) and ultimate tensile strength (UTS) gradually decreased. Then, the total elongation (TE) and UTS × TE (PSE) showed a trend of first increasing and then decreasing. The largest elongation of 54.9% and the highest PSE of 58.9 GPa% were obtained for sample AN550.

## 4. Discussion

### 4.1. Strengthening Mechanism

The yield strength of TWIP steels is influenced by lattice friction stress (*σ*_f_) and multiple strengthening mechanisms, such as the solid solution strengthening (*σ*_s_), grain refinement strengthening (*σ*_g_), dislocation strengthening (*σ*_d_), and precipitation strengthening (*σ*_p_). Due to the higher content of replacement atoms in high-manganese TWIP steels, the contribution of *σ*_s_ to the yield strength increment is less than 50 MPa [17,18,19]. Therefore, the *σ*_s_ value is neglected here. The contribution of various strengthening mechanisms to the yield strength can be calculated using Equation (1):

(1) $$∆\sigma_{y}=\sigma_{f}+\sigma_{p}+\sigma_{g}+\sigma_{d}$$

where *σ*_f_ is 250.7 MPa [5,20]. The contribution of *σ*_p_ to yield strength can be calculated using the following equation by simple calculation [21]:

(2) $$\sigma_{p}=\frac{0.538\;Gbf^{\frac{1}{2}}}{x}\ln\left(\frac{x}{2b}\right)$$

where G is the shear modulus (72.0 GPa); b is the Burgers vector (0.254 nm); and *f* and *x* represent the volume fraction and average size of precipitate, respectively (Table 1). The measured *σ*_p_ values of samples AN550, AN600, and AN650 are 147, 83, and 66 MPa, respectively.

The contribution of *σ*_g_ to yield strength can be estimated using the Hall–Petch equation [19]:

(3) $$\sigma_{g}=k_{y}d_{i}^{-\frac{1}{2}}$$

where *k*_y_ is constant, with a value of ~683.07 MPa µm^1/2^ [5], and *d*_i_ represents the average grain size. The calculated *σ*_g_ values for samples AN550, AN600, and AN650 are 185, 207, and 232 MPa, respectively.

According to Ref. [21], The contribution of *σ*_d_ can be used in the Taylor hardening law equation for evaluation.

(4) $$\sigma_{d}=M\alpha Gb\sqrt{\rho }$$

where M is the Taylor factor of 3.06 for FCC materials, α is the property constant of 0.25, and *ρ* is the dislocation density [18]. The average dislocation density (*ρ*) was calculated using Equation (5) [21]:

(5) $$\rho =\frac{\beta^{2}}{4.35\times b^{2}}$$

where *β* is the full width at half maximum (FWHM) of (111)**γ** diffraction peaks from X-ray diffraction patterns. The measured *ρ* value gradually decreased from 0.86 × 10^14^ in sample AN550 to 0.45 × 10^14^ in sample AN650 (Table 2). Subsequently, the calculated *σ*_d_ values for samples AN550, AN600, and AN650 were 129, 115, and 93 MPa, respectively.

**Figure 8:**
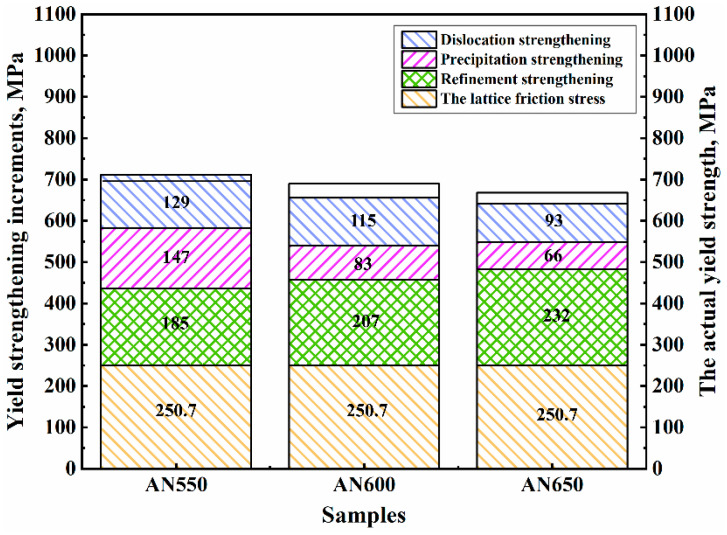
The contribution of the individual strengthening mechanism and the experimental yield strength.

Figure 8 shows the contribution of various strengthening mechanisms to the yield strength increments. The results show that the calculated values are similar to the experimental values. As the annealing temperature increased, the occurrence of recovery and the coarsening and dissolving of precipitate induced the decreases in ρ and *σ*_d_, respectively, while the activation of recrystallization further refined the grain and led to the increase in *σ*_g_.

### 4.2. Precipitates Affect Recrystallization Behavior

**Figure 9:**
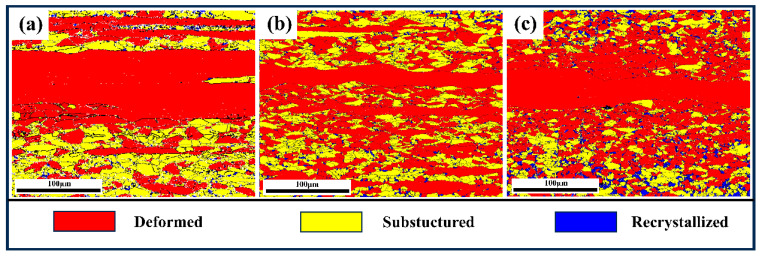
Recrystallized distribution maps of samples AN550 (**a**), AN600 (**b**), and AN650 (**c**), respectively. The red, yellow, and blue colors represented the deformed, partially recrystallized, and fully recrystallized grains, respectively.

Figure 9 shows the results of recrystallized microstructure analysis. Meanwhile, the red, yellow, and blue colors represent the deformed, partially recrystallized, and fully recrystallized grains, respectively. The fraction and size of recrystallized grain gradually increased with the increasing annealing temperature, while the average substructured grain size significantly decreased. This phenomenon was especially significant in sample AN650.

Simultaneous deformation during hot rolling leads to dense dislocation residues [5]. After annealing at a relatively lower temperature (550 °C), a relatively higher density of pre-existing dislocations (pre-dislocation) was retained in sample AN550. Due to the loose arrangement of atoms in the pre-dislocation region, the diffusion rate of atoms is much higher than that in the regular lattice, resulting in a faster atom diffusion rate [22]. The diffusion of atoms in the lattice is a thermally activated process that can be strongly accelerated using defects such as dislocations. This is ascribed to a reduced activation barrier [23]. The formation of cementite particles requires the diffusion of component elements at these dislocations through a pipeline mechanism [24,25]. Therefore, a higher pre-dislocation density in sample AN550 can ensure a faster solute diffusion rate and further provide more nucleation points for the cementite particles. In addition, the pre-dislocation structure includes numerous tangle dislocations, which increases the stability of the pre-dislocation structure. Subsequently, partial pre-dislocations recover during the annealing process. However, the early precipitated cementite particles can pin the pre-dislocations and hinder the recovery of dislocation, which further improves the stability of the dislocation structure. Specifically, fine carbides lead to the elimination of regions of high lattice misorientation, which are preferred sites for recrystallization nucleation, thus inhibiting recrystallization [24]. In addition, the presence of solute atoms and fine cementite particles hinders dislocation motion within the grain, thereby pinning sub-grain boundaries and preventing nucleation. Simultaneously, intergranular cementite particles impede grain boundary migration, thereby suppressing recrystallization, entailing solute drag and Zener pinning effects caused by precipitation [11,26]. This is because the diffusion rate of solute elements is lower than that of matrix atoms, resulting in solute atoms at the grain boundaries obstructing grain boundary migration and inhibiting grain growth [11]. Meanwhile, the finely dispersed precipitates provide greater resistance to grain boundary migration, leading to a more pronounced pinning effect and consequently reducing the rate of GB migration. The pinning pressure can be expressed by Equation (6) [27]:

(6) $$Pz=\frac{3F_{v}\gamma }{2r}$$

where *P*z is the pinning pressure, *F*v and r are the volume fraction and size of the precipitate, respectively, and γ is the boundary surface energy. The relationship between *P*_z_ and *F*v/r are observed to be proportional. Sample AN550 contains more fine precipitates (Figure 2b). According to Table 1, the *F*v/r values of samples AN550, AN600, and AN650 are 0.074, 0.031, and 0.022, respectively. Therefore, the finely dispersed precipitates exert greater resistance to GB migration in the AN550 sample. In addition, the shape of the precipitates also affects the pinning effect [11]. It was reported that the spherical precipitates were able to provide a higher pinning pressure [24].

In contrast, samples AN600 and AN650 were annealed at relatively high temperatures (600 °C and 650 °C). With the increase in annealing temperature, the continuous occurrence of recrystallization led to a decrease in dislocation density and a decrease in nucleation sites. However, a higher annealing temperature accelerates the diffusion of solute atoms to defects, and the solute atoms gather and even cause segregation at the GBs, leading to the coarsening and even dissolution of precipitates [26]. The consumption of solute atoms and the coarsening of precipitates weakens the drag effect and pinning effect [26]. The pinning pressure at GBs is specified in Equation (7) [28]:

(7) $$P_{GB}=\frac{F_{V_{GB}}\gamma D}{4r^{2}}$$

where F_VGB_ and D are the volume fraction and size of carbides at grain boundaries, respectively. P_GB_ is proportional to the F_VGB_/D ratio. According to Table 1, it can be inferred that an increase in annealing temperature leads to a gradual reduction in the pinning effect observed in AN600 and AN650 samples, thereby weakening the resistance to grain boundary migration and facilitating recrystallization (Figure 9b,c). In addition, it was reported that recrystallization preferentially nucleates at high orientation differences [24]. With the increase in annealing temperature, precipitates gradually dissolve and segregate, resulting in the uneven precipitation of carbides. The coarse particles interact with dislocation to provide the orientation gradient required for nucleation, which further facilitates recrystallization.

### 4.3. Deformation Mechanism and Strain-Hardening Behavior 

**Figure 10:**
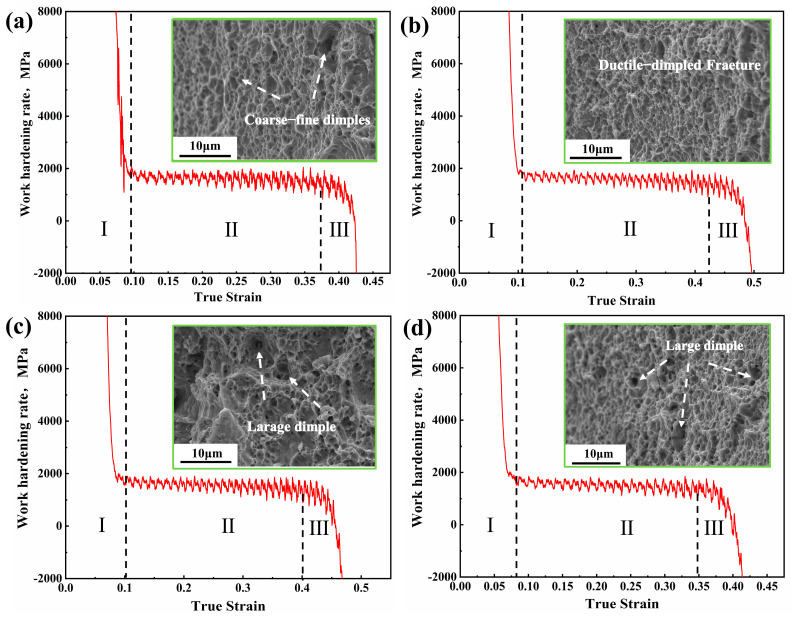
Work hardening rate (WHR) and fracture morphologies of annealed samples: (**a**) sample HR; (**b**) sample AN550; (**c**) sample AN600; (**d**) sample AN650.

The strain-hardening behavior was closely related to the deformation mechanism. Figure 10 shows the work-hardening rate-true strain curves and the corresponding fracture morphologies of all samples. All curves can be divided into three stages (I, II, and III). At stage I, the work hardening rate decreased rapidly due to the dynamic recovery of dislocations [14]. Then, the work hardening rates gradually reached constant values with the increasing true strain (stage II). Finally, the work-hardening rate significantly decreased until fracturing (stage III). The fracture morphologies of samples are shown in the top right corner of Figure 10a–d. All samples showed a large number of dimples. In contrast, the dimples were fine and evenly distributed in sample AN550 (Figure 10b), leading to the typical features of ductile fractures [14,29].

**Figure 11:**
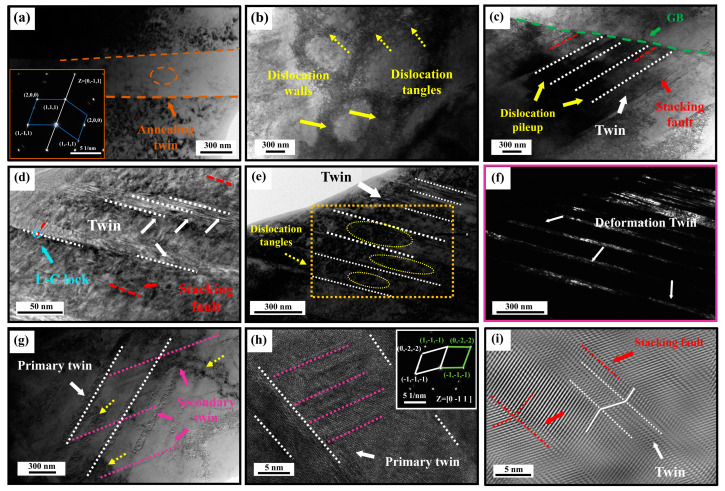
The interrupted tensile microstructures of sample AN550 under various true strains. (**a**) Before deformation. (**b**,**c**) At a true strain of 0.15, (**b**) dislocation tangles and dislocation walls; (**c**) stacking faults and twins near GB. (**d**–**f**) At a true strain of 0.3, (**d**) twin and L-C lock; (**e**,**f**) are the bright and dark field images, respectively. (**g**–**i**) After fracture, (**g**,**h**) are the bright field and HRTEM images of the initial and secondary twins, respectively; (**i**) is an inverse fast Fourier transform (IFFT) image.

Figure 11 shows the interrupted tensile microstructures of sample AN550 under various true strains. Recrystallization was inhibited by the precipitation of cementite in sample AN550 (Figure 9a). Before deformation, a high dislocation density was observed along both sides of the annealing twins, while the dislocation density inside was lower (Figure 11a).

At a true strain of 0.15, a number of dislocations were activated. The interaction between dislocations gradually forms dislocation tangles and several dislocation substructures, such as dislocation cells, as shown in Figure 11b. Simultaneously, a large number of dislocations accumulate at the GBs, due to the blocking effect of GBs, resulting in stress concentrations. In order to achieve release stress, grain boundaries become a source of dislocation radiation, resulting in rapid dislocation proliferation [30]. It was reported that the dissociation of pre-dislocation from partial dislocation can provide nucleation points for deformation twins and promote the formation of stratification faults [31]. Shockley partial dislocations were emitted to further promote the formation of stacking faults and twins at the GBs, enhancing WHR [30], as shown in Figure 11c. 

As the strain increases to 0.3, more dislocations are activated. A number of dislocations accumulate at the GBs, so that the stress reaches the critical stress required for the formation of twinning, thus promoting the generation of twins. In addition, the presence of a large number of dislocations moving near the stacking faults contributes to the formation of deformation twins [31]. Therefore, with the increase in strain, a large number of primary deformation twins are formed, causing continuous work hardening. Simultaneously, the interaction between stacking fault and deformation twin promotes the formation of the Lomer–Cottrell lock (Figure 11d), thus hindering the dislocation movement and enhancing the WHR [12,31]. In addition, the twin boundaries hinder dislocation motion, causing dislocation tangles. Further movement of the dislocation results in the formation of dislocation substructures between the twin lamellae (Figure 11e,f). However, the dislocation substructures can further impede dislocation movement, which promotes work hardening [23]. As strain increases, the formation of secondary twins can hinder dislocations from crossing the grain boundary barrier and avoid their annihilation, thus increasing dislocation density. Meanwhile, numerous deformation twins play a crucial role in grain refinement through the dynamic Hall–Petch effect (Figure 11g–i), thereby reducing the average free path of dislocations and further enhancing the WHR [1,32].

**Figure 12:**
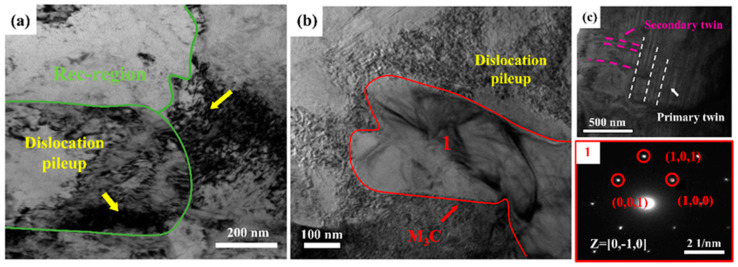
TEM images of sample AN650. (**a**) represents the microstructure prior to deformation, which displays a recrystallized structure; (**b**,**c**) are the microstructures after fracture; (**b**) shows the interaction between M_3_C and dislocations; (**c**) shows primary twins and secondary twins.

Figure 12a–c show the TEM images of sample AN650 before and after tensile deformation. The increase in annealing temperature weakens the pinning effect, thus promoting recrystallization and leading to a low dislocation density in the recrystallization region (Figure 9c and Figure 12a). The large number of recrystallized grains provides abundant space for the multiplication and accumulation of dislocations during subsequent plastic deformation [26]. As the strain increases, most of the strain is distributed to the softer recrystallized regions. To accommodate the strain gradient, long-range geometrically necessary dislocations (GNDs) are generated, thereby avoiding strain localization. Meanwhile, continuous back stress hardening is provided, which contributes to the WHR [23]. In addition, precipitate particles strongly inhibit dislocation movement during the straining process, further increasing WHR (Figure 12b).

However, the ductility of samples AN600 and AN650 deteriorated compared with sample AN550. An increase in annealing temperature induces the coarsening of precipitates, especially the particles in GBs (Figure 2a–c). On one hand, the large-sized carbides at the interface can increase the stacking fault energy at GBs, which increases the critical stress for twin generation, thereby lowering ductility. On the other hand, the coarse intergranular precipitate changes the lattice parameters of austenite. It is easy to produce stress concentrations to form vacancies that evolve into crack sources [33,34,35], further lowering ductility. In addition, the uneven precipitation of coarse carbides can cause local stress concentrations due to the pile-up of slip bands, further compromising ductility.

### 4.4. Microstructural Evolution

**Figure 13:**
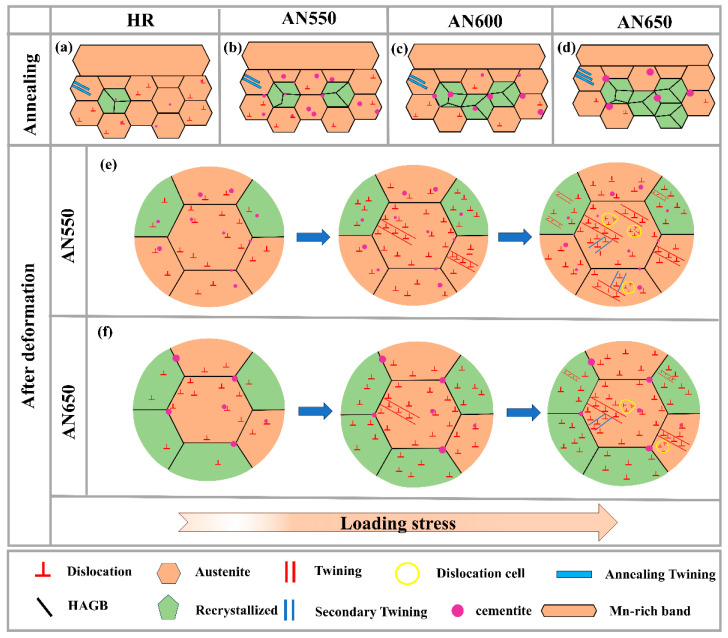
Microstructural evolution diagrams of different samples after annealing (**a**–**d**), and after deformation for AN550 and AN650 samples (**e**,**f**). (**a**) HR; (**b**,**e**) AN550; (**c**) AN600; (**d**,**f**) AN650.

Figure 13 displays the abstract microstructure evolution of annealed samples before and after tensile deformation. With the increase in annealing temperature, the size of cementite particles gradually coarsened in samples HR, AN550, AN600, and AN650. The volume fraction of cementite particles first increased and then decreased. The volume fraction of recrystallization gradually increased. In contrast, sample AN550 has the highest volume fraction of cementite and a relatively high dislocation density (Figure 13a–d).

In the process of tensile deformation, multiple deformation mechanisms were generated (Figure 13e,f). The high-density dislocations activate the TWIP effect in all samples. As the dislocations in the recrystallization zone are relatively even, they facilitate the formation of thinner deformation twins. With the increase in tensile strain, secondary twins are activated, and the formation of substructures is promoted (Figure 13e,f). As the strain must be continuous at the connection interface, GNDs are produced to ease the strain gradient and avoid the formation of strain gradients in the vicinity of the interface [23]. In the AN550 sample, the volume fraction of partial recrystallization is elevated. Meanwhile, the pinning effect of an appropriate quantity of cementite particles with relatively small sizes on grain boundaries dislocations is significantly increased, which significantly promotes the dislocation storage energy (Figure 13e). For sample AN650, even though the volume fraction of recrystallization is further enhanced, a large number of coarsened cementite particles are distributed along the grain boundaries, resulting in the deterioration of ductility.

## 5. Conclusions

In this study, the hot-rolling annealing process tailors the precipitation of cementite particles. Cementite influences recrystallization behavior. The conclusions are as follows:

1. Higher-density pre-dislocation promotes the precipitation of fine cementite particles, resulting in higher pinning pressure. With the increase in annealing temperature, the coarsening of cementite particles weakens the pinning pressure.
2. An appropriate amount of fine cementite particles results in a relatively low volume fraction of recrystallization in sample AN550, which shows comprehensive mechanical properties with a YS of 701 MPa and a PSE value of 58.9 GPa%. Precipitation and dislocation strengthening play an essential role in YS.
3. Multiple deformation mechanisms of dislocation, stratification, primary twin, and secondary twin interact with each other, providing continuous work hardening for sample AN550.

## Figures and Tables

**Table 1:** The volume fraction and average grain (*d*)/particle (*x*) size in the samples.

Date	HR	AN550	AN600	AN650
Average particle size(*x*)/nm	60	85	145	180
Volume fraction(*f*)	0.020	0.063	0.045	0.039
Average grain size(d)/μm	15.8	13.8	10.8	8.5

**Table 2:** Full width at half maximum examined along the RD and the dislocation density for austenite.

Samples	FWHM (rad)	Ρ (m^−1^ × 10^14^)
	γ(111)	
AN550	0.004905946	0.86
AN600	0.004355295	0.68
AN650	0.003540051	0.45

## Data Availability

The original contributions presented in this study are included in the article. Further inquiries can be directed to the corresponding authors.
